# Detection and Plant Monitoring Programs: Lessons from an Intensive Survey of *Asclepias meadii* with Five Observers

**DOI:** 10.1371/journal.pone.0052762

**Published:** 2012-12-20

**Authors:** Helen M. Alexander, Aaron W. Reed, W. Dean Kettle, Norman A. Slade, Sarah A. Bodbyl Roels, Cathy D. Collins, Vaughn Salisbury

**Affiliations:** 1 Department of Ecology and Evolutionary Biology, University of Kansas, Lawrence, Kansas, United States of America; 2 School of Biological Sciences, University of Missouri-Kansas City, Kansas City, Missouri, United States of America; 3 Kansas Biological Survey, University of Kansas, Lawrence, Kansas, United States of America; 4 Natural History Museum/Biodiversity Research Center, University of Kansas, Lawrence, Kansas, United States of America; 5 Department of Biology, Colby College, Waterville, Maine, United States of America; University of Alberta, Canada

## Abstract

Monitoring programs, where numbers of individuals are followed through time, are central to conservation. Although incomplete detection is expected with wildlife surveys, this topic is rarely considered with plants. However, if plants are missed in surveys, raw count data can lead to biased estimates of population abundance and vital rates. To illustrate, we had five independent observers survey patches of the rare plant *Asclepias meadii* at two prairie sites. We analyzed data with two mark-recapture approaches. Using the program CAPTURE, the estimated number of patches equaled the detected number for a burned site, but exceeded detected numbers by 28% for an unburned site. Analyses of detected patches using Huggins models revealed important effects of observer, patch state (flowering/nonflowering), and patch size (number of stems) on probabilities of detection. Although some results were expected (i.e. greater detection of flowering than nonflowering patches), the importance of our approach is the ability to quantify the magnitude of detection problems. We also evaluated the degree to which increased observer numbers improved detection: smaller groups (3–4 observers) generally found 90 – 99% of the patches found by all five people, but pairs of observers or single observers had high error and detection depended on which individuals were involved. We conclude that an intensive study at the start of a long-term monitoring study provides essential information about probabilities of detection and what factors cause plants to be missed. This information can guide development of monitoring programs.

## Introduction

Conservation biologists use long-term monitoring to characterize population trajectories, quantify rates of survival and fecundity, and explore how population data relate to management. When collected with reliable protocols, such data provide essential information for decision-making in applied ecology. With few exceptions, population monitoring ultimately depends on observers' records of the number of plants and animals in field environments. Counting organisms can, however, be challenging: organisms may be mobile or cryptic, observers may vary in their detection skills, and often survey time is limited. Conservation biologists therefore need to explicitly consider detection issues in field protocols and data analysis [1]–[3]. Many zoologists do use methods that provide estimates of population size and vital rates despite incomplete detection [4]–[6]. Incomplete detection of plants, however, has received less attention. Although sessile plants are likely inherently easier to study than animals, imperfect detection can result from processes that make it impossible or unlikely to observe plants (i.e. growing-season dormancy, seed banks, herbivory prior to surveys) and from observer error [2], [7]–[12]. Ignoring incomplete detection can lead to bias in estimating plant population distributions, sizes, survival and recruitment rates, as well as population growth rates and extinction probabilities [7], [10], [12]–[16].

We illustrate how an intensive study at the start of a plant monitoring program can 1) identify whether probabilities of detection are less than one and, 2) if so, quantify what factors affect detection. We focus on observer error, which we define as cases where people do not observe plants that have above-ground parts at the time of the survey. Quantifying observer error typically involves a “double observer” methodology (i.e., the presence or absence of plants is surveyed independently by at least two observers (or one observer at two time periods)). If results differ between the two surveys, detection probabilities can be estimated using closed mark-recapture models (which assume no changes in the population size over the survey period). Past work suggests many reasons for observer error. For example, in contrast to the near 100% detection of flowering orchids, the probability of initial detection of vegetative plants was approximately 0.8 [17], [18]. In a Chinese forest, detection of the presence or absence of different trees and shrubs varied from 0.09 to 0.34 [11]. Variation in detection among observers can also be important: detection probabilities varied from 0.09 to 1.0 among 12 observers searching for an exotic plant [12].

A practical challenge with double observer methodologies is that repeated visits to plants can lead to vegetation tramping. Trampling can have two adverse effects. First, plant growth, herbivory rates, and soil properties can be altered by repeated visits to individual plants [19]–[21]. Second, trampling complicates interpretation of mark-recapture analyses. Specifically, the probability of detection is likely to increase between first and subsequent observers because later observers may notice paths that the first observer took in finding plants. These negative effects of trampling are expected with dense herbaceous vegetation (i.e. prairies, savannahs), which are exactly the habitats where imperfect detection is problematic [13], [22].

Our work focuses on Mead's milkweed (*Asclepias meadii* (Torr ex A. Gray)), a rare prairie plant. In past studies, we found detection probabilities considerably less than one, and utilized this information to improve estimates of population size, survival rates, and population growth rates [10], [13], [14], [23]. As we expanded our work to new sites we faced the classic challenge: how does one accurately determine the numbers of plants in a defined area within a reasonable amount of time? Our solution was an intensive survey that quickly provided data on imperfect detection, yet avoided vegetation trampling. In our analyses, we estimated the total number of patches (our surrogate for individual plants) for study areas at two sites. For detected patches, we examined whether detection was affected by a) patch state (flowering or not flowering), b) patch size (number of stems), c) patch distance (near or far from observer) and d) observer (four experienced observers and one naïve observer). Finally, we evaluated how the number of observers affected the probability of a patch being detected by at least one person. Although our specific results are restricted to this species, sites, and group of observers, our general approach illustrates explicit consideration of detection in monitoring, and thus has broad applications for plant conservation biologists.

## Methods

### Study species

*Asclepias meadii* (Asclepiadoideae, Apocynaceae) is a long-lived herbaceous perennial of tallgrass prairies and glades in Kansas and Missouri, USA and is listed as threatened under the Endangered Species Act [24], [25]. Plants consist of one to many flowering and nonflowering stems, with underground rhizome connections. Flowering stems have an average of twelve white flowers in early summer [26]; fruits are produced in late summer. At the Anderson County Prairie Preserve (hereafter, Preserve), flowering and nonflowering stems are typically 27 to 40 cm and 20 to 25 cm tall, respectively. See [27] for details of the species' ecology.

### Study sites

The Preserve is a 554 ha area of conservation significance in eastern Kansas (USA), 8 km south of Garnett, KS; of the 282 known locations of *A. meadii*, this site is listed as the largest population (478 stems in 2001; K. Lah, 2008 compilation, personal communications). 84% of the other known locations have 30 or fewer stems. We used units 9 (6.9 ha) and 10 (20.2 ha) of the Preserve. Both units are unplowed native tallgrass prairie that had been hayed in mid summer since at least the 1940′s. Divergent management began in 1999: unit 9 was hayed from 1999–2006 (with no burns), whereas Unit 10 was not hayed and burned only in 2001. On 16 April 2007, unit 10 was burned again. The fire in unit 10 (hereafter, burned site) created open bare patches, but also regrowth of grasses. Unit 9 (unburned site) had few bare patches. The Preserve is owned by the Nature Conservancy and managed by the University of Kansas Field Station. We had approval of the Station for this work.

### Field methods

On 22–23 May 2007, we established three 100 m×4 m transects in both burned and unburned sites; each 100 m transect had markers at 20 m intervals. Within each site, transects were separated by at least 8 m. Logistical issues made it impossible to choose strictly random locations for the transects, as is most desirable [1], but care was taken to choose locations that were typical of the larger area. Importantly, transects were established without regard to locations of *A. meadii*. On 4 June 2007, we laid out 100 m tapes, and five individuals (observers A–E) independently searched for stems of *A. meadii* along each transect. Four of the five observers had experience with *A. meadii* surveys (A–D). The naïve observer (E) was trained in field identification of *A. meadii* for approximately one hour. Individuals were instructed to record the time spent searching within each 20 m portion of each transect, with the goal of completing a transect in 40 minutes (8 minutes per 20 m portion). Each individual walked with feet close to, and on either side of the meter tape and did not walk into the surrounding vegetation. He or she then visually searched a 2 m area to the right and left of the meter tape. When a stem was found, its state (flowering vs. nonflowering) and x,y coordinates were recorded. Coordinates were defined as y =  location along the meter tape, and x =  distance to the right or left of the meter tape as measured by a placing a 2 m pole (marked in 5 cm intervals) perpendicular to the tape with the 0 end located at the tape. Our method ensured independent detection by each observer: each person worked alone and we did not trample vegetation around stems which could have provided insight on stem locations to others. The immediate area next to the tape was, of course, trampled but this small area rarely contained *A. meadii* stems. Next, the x,y coordinates from all five observers were plotted on maps so that a subset of researchers could verify the location of all stems on 5–6 June. Very few errors were found, but this reconciliation step corrected cases where people recorded data to the right instead of the left of the central transect line (or vice versa) or misidentified the species. This validation was important since these rare errors could lead to patches with single detection encounter histories (see below), artificially decreasing detection probabilities.

We defined “patches” of plants using the criterion that stems in the same patch were separated by no more than 1.25 m. This definition was used in past work [10], [13], [14], [27] and was chosen because the maximum length of rhizomes observed in this species is 1 m (Marlin Bowles, personal observation). Patches are likely genetic individuals, but we use “patch” to be conservative since seeds falling from fruits could potentially germinate within the mother plant [27]. Patches often consisted of stems separated from each other by 5 to 30 cm; the largest patches had multiple stems within a 1–2 m^2^ area. We did remove one area (30 m – 40 m, transect 1) from analyses because milkweed stems were scattered throughout, hindering clear patch designations. Hence our study area was 2360, not 2400 m^2^.

Each patch was assigned as flowering (at least one flowering stem) or nonflowering (no flowering stems) and patch size was defined as the maximum number of stems per patch found by all observers. We recognize that patches close to the transect edge were not completely surveyed; thus, in some cases, patch size reflects the size of the patch within our survey area and underestimates actual patch size. Patch distance was classified as either near (≤1 m from the measuring tape) or far (>1 m from the measuring tape). If patches spanned the 1 m mark and were flowering, we assigned “near” vs. “far” by the location of the flowering stem, given our past experience that flowering increases detection [10], [14]. If a patch had only nonflowering stems, we assigned “near” vs. “far” by averaging the x coordinate for all stems per patch, with weighting based on the number of observers detecting each stem. Observers were noted to have detected a patch if he or she found at least one stem in the patch. We assigned encounter histories for each patch; a history consisted of five digits, where 0 =  no detection and 1 =  detection and each digit refers to an observer. For example, a patch with an encounter history of 10011 was detected by observers A, D, and E.

### Analyses

We created two encounter history data sets, one for each site (there were too few patches per transect to do transect-specific analyses). First, using the program CAPTURE [28], we estimated the total number of patches (in the set of three transects at each site) based on the encounter histories for each site. Typically encounter histories describe detection or lack of detection of individuals over multiple time periods, so our five “observers” are analogous to five “time periods” in most mark-recapture applications. CAPTURE can account for variability in probability of detection: we focused on “temporal” variation (variation in detection among the five observers; typical notation of M_t_ has been replaced by M_obs_ in this paper) and “heterogeneity” (differences in detection among patches). We did not consider CAPTURE models with “behavioral” variation since this would imply that detection varied depending on observer order; our methods were designed for independence of observers (i.e. avoiding vegetation trampling). We focused on four possible models: M_o_ (no variation among observers, homogeneity of detection among patches), M_obs_ (only observer variation), M_h_ (only heterogeneity in detection among patches), and M_obs,h_ (observer variation, heterogeneity in detection among patches). We ran separate models for the burned site (flowering), burned site (nonflowering), and unburned site (nonflowering) because past work revealed that the presence/absence of flowering stems was important in patch detection [10], [14]. We did not run a unburned (flowering) model because only 3 flowering patches were found at that site. To choose the best model for each data set, we used model selection procedures within CAPTURE [29].

Next, we used the Huggins model [30] within the software package MARK [31] to focus on detected patches; this model uses covariates to determine what variables are predictors of incomplete detection. We defined sites as groups and considered patch state (flowering vs. nonflowering), patch size (number of stems), and patch distance (near vs. far) as individual covariates. The typical “temporal” effect in this model was, as in CAPTURE, a measure of variation in detection among observers A–E. The program assumes multiple sampling times and encounter histories are interpreted in terms of initial and subsequent detection; this was inconsistent with our study. We thus analyzed the data by assuming that 100% of patches were initially detected (by adding a “1” at the start of all encounter histories); the five “resighting probabilities” estimated by the program were therefore the initial detection probabilities for observers A – E. With three covariates and potential observer differences, it was not feasible to run all possible models. Hence, one of us (AWR) explored subsets of all possible models, with more model variants utilizing a particular covariate if previous models that included that covariate had high fit. We used Akaike's information criterion (AIC_c_) values to select the most parsimonious models from the set of explored models. Following [32], the best model had the lowest AIC_c_ score. Models with similar scores (AIC_c_ <2) were considered to have similar support, and we used model weights for averaging estimated parameters among these models. To average models, we ran each model with specified values for each individual covariate. We then used the parameter estimates for resighting probability and the model weight to derive model-averaged estimates for each parameter. This process was repeated for each value and combination of individual covariates. We estimated abundance by dividing count data by probabilities of detection [33]; specifically, we divided the number of patches seen for combinations of site, observer, patch state, and patch size by model-averaged probabilities of detection for these same combinations of factors. To illustrate the impact of ignoring a source of variation in detection, we compared estimated abundance using probabilities of detection from a reduced model that ignored patch size.

**Figure 1:**
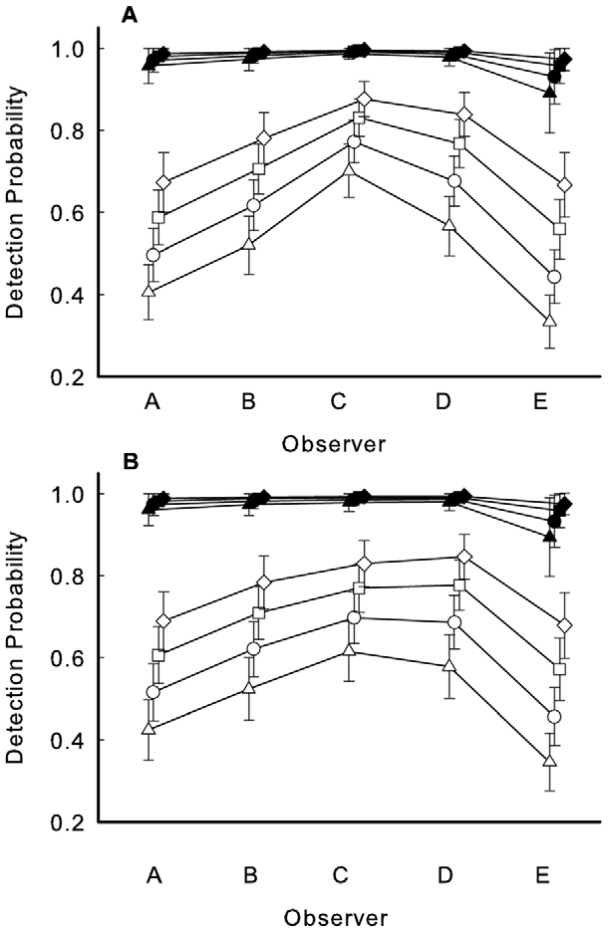
Probabilities of detection for observers A – E calculated from a Huggins model. Probabilities were calculated for flowering (filled symbols) and nonflowering (open symbols) patches with 1 (triangle), 2 (circle), 3 (square) and >4 (diamond) stems per patch for a) burned and b) unburned prairie sites. Symbols are offset so that SE values can be examined. Lines connect values for the same patch state and size for different observers.

For each site, we also determined group probabilities of detection; i.e. *p_5_ = * the probability that a patch was detected by at least one of the five observers, assuming independence of observers. This value was calculated as:
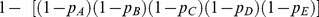
(1)where *p_A,_ p_B,_ p_C,_ p_D,_* and *p_E_* refer to observer-specific detection probabilities. We calculated a common variance using the delta method [34]. Each observer-specific detection probability was the average of the observer's probability of detection for patches of different types (i.e. patch state and size, see Fig. 1), with weighting by the number of patches detected by all five observers at each site. We also explored the effect of number and identity of observers on detection. Specifically, we determined the probability that a patch was detected by all possible combinations of groups of two, three, or four observers (e.g., *p_2_, p_3_, p_4_*). Given that detection of nonflowering patches is more challenging, we also calculated {*p_A,_ p_B,_ p_C,_ p_D,_ p_E_* } and {*p_2,_ p_3,_ p_4,_* and *p_5_*} for only nonflowering patches at each site.

## Results

### Overview

The five observers detected 51 (8 flowering, 43 nonflowering) and 35 (3 flowering, 32 nonflowering) patches in the burned and unburned sites, respectively ( Table S1). For detected patches, the proportion of flowering patches did not differ significantly between sites (Fisher Exact Test, *P* = 0.51); we thus combined patches from both sites to compare numbers of stems for flowering vs. nonflowering patches. The median number of stems per patch was higher for flowering patches (3, range 2–9) than for nonflowering patches (2, range 1–12) (Kruskal-Wallis test, *H* = 6.76, d.f. = 1, *P = *0.01). After partitioning patches into one versus two or more stems, we found 44% of nonflowering patches had a single stem compared to 0% of flowering patches (Fisher Exact Test, *P = *0.006). All but one detected flowering patch were seen by all observers; the percentage of detected nonflowering patches seen by each observer was variable and depended on patch size (Table S1).

Each observer was asked to spend 40 minutes per transect. These instructions were largely followed in the burned site but there was more variation in survey time at the unburned site (Table S1). Although the observer spending the longest time had highest detection probability at each site (see below), overall there was no clear relationship between the proportion of detected patches that an observer saw and the time spent surveying.

### Mark-recapture analyses

For the burned site, M_o_ and M_obs_ were the best fitting CAPTURE models for the flowering and nonflowering patches, respectively. Estimated population sizes were 8 for flowering patches (SE = 0.0003) and 43 for nonflowering patches (SE = 0.0109); these estimates exactly matched the number of detected patches of each type. For the unburned site, M_h_ was the best fitting model for the nonflowering patches, with an estimated population size of 41 (SE = 5.34); the 95% confidence interval for numbers of patches did not include the number of nonflowering patches detected at this site (32).

Using the Huggins model, the best fitting model (model 1) revealed that probability of detection was higher for flowering patches relative to nonflowering patches, and detection increased with increasing number of stems per patch. Observers also differed in probability of detection (Table 1). Three other models also fit the data well (models 2, 3, 4; Table 1). We thus used model averaging to estimate probability of detection for combinations of observers, patch state, and patch size for each site (Fig. 1; we did not include patch distance since it was a factor only in the fourth model). Model 2 was similar to model 1 but included an interaction so that the overall detection probability of observers depended on the site (compare Observer C and D at the two sites, Fig. 1). In model 3, one or more observers differed in how the presence or absence of flowering or number of stems affected their detection (suggestion of nonparallel lines in Fig. 1, implying the number of stems per patch affected detection by some observers more than others). Finally, model 4 was similar to model 1 but suggested that patch distance (near/far) affected detection. Although near vs. far had no apparent effect for observers A-D, observer E had slightly higher detection for “far” nonflowering patches with 1 or 2 stems. When we incorporated model-averaged detection probabilities due to site, state, observer, and patch size, our estimated abundances were similar to the observed number of detected patches (Table S1). When we ignored variation due to patch size, our estimated abundances were lower than observed numbers (Table S1).

**Table 1:** Comparison of Huggins models.

	AIC_c_	Delta AIC_c_	*w_i_*	k	Dev	State	Size	Dist	Site	Obs	Interactions
M1	476.8	0.00	0.38	7	462.5	-	-			-	
M2	478.3	1.54	0.18	12	453.7	-	-				Site*Obs
M3	478.5	1.71	0.16	11	455.9	-	-			-	State*Obs, Size*Obs
M$	478.7	1.90	0.15	8	462.4	-	-	-		-	

The four best fitting models are shown (M1–M4), with model 1 having the lowest AIC_c_ value and thus the best fit. Dev describes the fit of the model, k is the number of parameters and *w_i_* refers to the weighting factor. A dash indicates whether a model included a term for differentiating probability of detection depending on patch state (flowering vs. nonflowering), patch size (number of stems), patch distance (dist; near or far from observer), site (burned vs. unburned), and observer (obs; individuals A–E). Interaction terms are noted.

**Figure 2:**
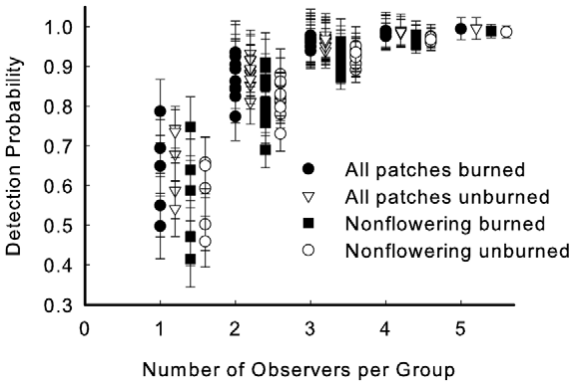
Probability of detection of patches depending on the number of observers per group. Numbers of observers per group range from 1 – 5; probabilities shown are *p_1_, p_2_, p_3,_ p_4,_* and *p_5_*, defined as the probability that at least one observer in a group of defined size will detect patches; see text). For each group size, probabilities are indicated for four categories (all vs. only nonflowering patches, burned vs. unburned site). For group size 5, a single detection probability was calculated for each category (see equation 1). For group sizes 2–4, probabilities of detection are indicated for all combinations of the number of observers (10 combinations for 2 and 3 observers, 5 combinations for 4 observers; see text). For group size 1, five values are shown, corresponding to the observer-specific detection probabilities for the five observers in the actual study. Bars are SE of a common variance.

If we considered observer-specific probabilities of detection for the five observers, the probability of detection of a patch was very high (*p_5_*>0.98), regardless of site and whether all patches or only nonflowering patches were included (Fig. 2). If surveys had been done with fewer observers, our analyses suggest overall detection would decrease and be increasingly affected by the particular combination of observers (Fig. 2).

## Discussion

### Plant monitoring challenges

We asked a deceptively simple question: how likely is it that all individuals of a species of interest will be detected in a single survey? This question is central for many conservation goals, including monitoring of rare [10] and exotic species [12], [35]. Typically, estimation of plant population size involves counting individuals in small plots or diverse plot-less methods [36]–[38]. These methods can be very successful; detection issues are not a concern for all species. However, if characteristics of the plant species, site, or observers reduce detection, expensive and time-intensive field work may lead to erroneous conclusions, even when performed by skilled observers. Not only can total population sizes be underestimated, but we may misinterpret population structure (i.e. overestimate the abundance of larger or flowering individuals). If the goal of the survey is to initiate a long-term demography study, incomplete detection could lead to such work being done on a nonrandom subset of the population. These issues are not new: field biologists know they can miss plants and recent work documents the extent of detection problems across species [11]. However it has been challenging for plant biologists to go from general knowledge of the issue to incorporating detection in their own work. Further, although we applaud the momentum behind citizen science, involving more naïve observers will likely increase the need to consider detectability to ensure data quality [39].

Given that time, budgets, and human resources are inevitably in short supply in monitoring, we limited our primary data collection to a single day, but worked at two sites with five observers. Our results gave us confidence that flowering patches will be found, even by single observers. However, we were sobered by the low probabilities of detection for nonflowering patches, the importance of stem number in patch detection, and the differences in detection among observers, especially since four of the five people had extensive experience with this species. Observers A and B, for example, had participated in surveys of *A. meadii* for over 10 years. However, observers C and D detected more patches yet had less experience (1–3 prior surveys). Individual E is an accomplished ecologist but had never worked with the species. An observer's detection level also may depend on the degree to which he/she tried to finish a transect within the 40 minute guideline. Further, we note that although the positive relationship between probabilities of detection and number of observers was expected, it was striking that three or more observers were needed to have consistently high detection and probabilities greatly depended on observer identity. Two points should be emphasized. First, no observers were redundant: everyone saw some patches that were not seen by others. Second, even though combinations of two observers did not yield close to 100% detection, use of two observers is greatly preferred over a single observer since it allows estimation of probability of detection, and thus quantifies the extent of the problem. Our work at two sites allowed us to begin exploring the generality of our approach. Given little evidence of site-specificity (similarity of Fig. 1a,b), our methodology may be effective for monitoring across haymeadow sites, the most common habitat for this plant.

There is no point in doing a “double observer” survey if the second observer discovers plants by simply following the trampled vegetation from the first observer. To ensure independent discoveries of plants by multiple observers, we developed a protocol using distance sampling methods [40] which have only rarely been applied to plants [41]. A surveyor using this approach typically walks along a defined line and records the estimated distances to detected individuals (measuring perpendicular to the line). In wildlife applications, the observed decline in number of animals detected at increasing distances from the line is central to estimation of population size. With *A. meadii*, we found no evidence of reduced detection at increasing distance (i.e. “near” plants were not more likely to be seen). The two meter survey area on either side of a transect line is thus an appropriate distance for *A. meadii* detection with our field conditions. Using the 4 m×100 m belt transect approach also allowed our crew to move quickly across the landscape. Covering relatively large areas was essential since *A. meadii* is scattered across prairies and the units of interest are not only single stems but also defined patches of stems (presumably genotypes [27]).

### Analyses of plant abundance and probabilities of detection

Prior to intensive analysis of detection, one can simply examine data from double observer surveys: if the plants found by the first observer were inevitably found by a second observer, there is no cause for concern. However, if detection issues are evident, mark-recapture programs should be employed. We took a two-step approach. First, we addressed whether our surveys likely detected all visible patches. We estimated abundance taking into account variation in detection among observers and patches (eliminating variation due to patch state by subdividing the data set). For the burned site, CAPTURE results suggest that the five-person group found all patches. However, for the unburned site, the number of estimated nonflowering patches was 28% higher than the number detected. CAPTURE model M_h_ was selected for the unburned site, indicating that patches differed in likelihood of being found. This heterogeneity in detection may be due to the large variation in patch size (equal numbers of detected patches with very small or very large numbers of stems, Supplementary Table S1) as well as likely variation in stem height. The net result was that even surveys by five observers likely missed patches.

Our second step focused on the detected patches. At both sites, variation in detection due to patch state (flowering/not flowering), patch size (number of stems), and observer (individuals A – E) was evident. All five observers in our survey had high detection of flowering patches, but there was considerable variation in detection of nonflowering patches (Fig. 1). The naïve observer (E) did not appear to be greatly handicapped by lack of experience. This result is not generalizable (i.e. only one naïve observer was used); other studies with multiple observers have [42] or have not [12] demonstrated effects of observer experience on detection.

Search time also could also affect detection. The 40-minute time span per transect was chosen because it allowed surveyors to search for plants at a slow but steady pace and complete all six transects in a single day. Conservation monitoring in general is time-limited: monitoring typically requires workers to visit many remote sites, and thus time per site has defined bounds (see [42] for related discussion on floristic surveys). Detection probabilities are also, of course, dependent on season and habitat: we surveyed in early summer to maximize detectability (i.e. *A. meadii* is flowering; prairie grasses are relatively short).

A major strength of the Huggins mark-recapture model [30] is that it incorporates covariates so causes of incomplete detection can be explored. However patches that are not detected cannot be assigned covariates. To deal with this problem, Huggins used conditional likelihood theory; he conditioned the model on only the patches that were detected. Therefore, the model assumes that detected and undetected patches are the same in terms of their state, size, and distance from the observer ([43], http://warnercnr.colostate.edu/class_info/fw663/Mark.html). In the case of flowering patches, CAPTURE analyses suggest that this assumption was not a concern: we saw all reproductive individuals. For nonflowering patches, we had complete detection at the burned site but not at the unburned site. We suspect that single-stemmed nonflowering patches have been missed at the unburned site, potentially making the effect of patch size on detection even more pronounced than evident in Figure 1. However, our CAPTURE analysis was performed separately for the two sites whereas the Huggins model was allowed to choose the best models regardless of site. Detection probabilities in the burned site thus may be less than one and perhaps overestimated by the CAPTURE analysis.

## Conclusions

In our work on *A. meadii,* we had hoped that five observers would prove unnecessary for monitoring. However, a five observer team did improve our estimates of numbers and thus we retained them in our monitoring. Our next goal is to use multiple years of data to estimate vital rates (i.e., patch survival). Extremely high survivorship seems necessary to explain the persistence of haymeadow populations of *A. meadii* because complete removal of fruiting stems with haying means seedling recruitment must be close to zero. We want to compare patch survival rates at the haymeadow sites to the 0.95 survival rate found at a periodically burned site [10]. *Asclepias meadii* may respond to haying by more extensive vegetative growth [24], [44], thus spreading mortality risk among multiple stems.

The strength of our study is its exploration of detection while considering multiple observers, plants of different states/sizes, and two sites (and avoiding vegetation trampling). Other recent studies explore plant detection probabilities from other perspectives [11], [12], [45], [46]. Regardless of the specific approach, three classes of questions are important to address as one embarks on a survey. First, the observers themselves: do observers vary in ability? experience? motivation? Second, the target plants: how does plant species, size, or stage (seedling, nonflowering plant, flowering plant) affect detection? Third, the habitat: does detection differ among sites? depend on habitat structure, relative size of the target plants versus background vegetation, or frequency of encounter of the target plants (which may alter both observer detection and motivation)? All three issues may change with weather or season. Given the diversity of situations, we do not advocate a single universal sampling protocol. Instead, we encourage botanists to quantify diverse factors that may affect detection at the start of studies, and to utilize this information in subsequent development of monitoring programs. Given species differences in detectability, this general issue is equally relevant for research questions at the community level [11].

## Supporting Information

Table S1 **Number of patches detected by each observer according to state (flowering or nonflowering) and patch size (number of stems) in burned and unburned prairies.**
(DOCX)
