# Supplementary material for: Detection and Plant Monitoring Programs: Lessons from an Intensive Survey of Asclepias meadii with Five Observers
Source: PLoS One. 2012 Dec 20;7(12):e52762. doi: 10.1371/journal.pone.0052762 (PMC3527611; doi:10.1371/journal.pone.0052762)
Supplement: Table S1 — Number of patches detected by each observer according to state (flowering or nonflowering) and patch size (number of stems) in burned and unburned prairies. (DOCX) [file pone.0052762.s001.docx]

**Table S1.** Number of patches detected by each observer according to state (flowering vs. nonflowering) and patch size (number of stems) in A) burned and B) unburned prairie sites in Anderson County, Kansas (USA). For each combination of patch state and size, the total number of detected patches an observer saw is shown. The total number of observed patches for each combination of observer, patch state, and patch size divided by probability of detection (using model-averaged values in Fig. 1) provides an estimate of total patch number (listed in parentheses after count data, with 95% confidence intervals in square brackets below estimates unless upper limit – lower limit = 0). Such estimates were not calculated when observed numbers = 0, or for the unburned flowering data where only three patches were seen by any observer. The ‘all patches’ rows indicate the total number of patches seen by an observer for combinations of patch state and site, with the estimated abundance from a reduced model that ignored patch size in parentheses (95% confidence intervals are in square brackets below estimates). The total time (minutes) each observer spent searching for plants at each site is also indicated (sum of times for the 3 transects per site). ____________________________________________________________________________________________________________

A. Observer: A B C D E All observers

Flowering patches

1 stem 0 0 0 0 0 0

2 stems 4 (4.1) 4 (4.1) 4 (4.0) 4 (4.1) 4 (4.3) 4

[4.8,4.0] [5.3,4.0] [4.6,4.0) [5.0,4.1]

3 stems 2 (2.0) 2 (2.0) 2 (2.0) 2 (2.0) 1 (1.0) 2

> 4 stems 2 (2.0) 2 (2.0) 2 (2.0) 2 (2.0) 2 (2.1) 2

[2.2,2.0]

All patches 8 8 8 8 7 8

Nonflowering patches

1 stem 10 (24.7) 13 (25.0) 20 (28.5) 14 (24.7) 10 (30.0) 23

[42.1,16.7] [37.2,19.0] [34.7,24.9] [34.8,19.5] [46.1,21.1]

2 stems 6 (12.1) 7 (11.3) 8 (10.3) 6 (8.9) 3 (6.8) 11

[25.0,8.0] [19.9,8.4] [15.0,8.8] [13.1,7.2] [9.8,5.1]

3 stems 2 (3.4) 3 (4.2) 6 (7.2) 6 (7.8) 3 (5.4) 7

[3.8,3.3] [4.5,4.0] [8.8,6.5] [10.6,6.7] [6.8,4.5]

> 4 stems 1 (1.5) 2 (2.6) 2 (2.3) 1 (1.2) 2 (3.0) 2

[2.0,1.2] [1.4,1.1] [3.1,2.9]

All patches 19 (35.9) 25 (38.6) 36 (41.7) 27 (39.3) 18 (36.7) 43

[43.0,30.9] [43.0,35.3] [43.0,40.7] [42.9,36.5] [44.0,31.5]

Time (min.) 108 118 124 119 111

B. Observer: A B C D E All observers

Flowering patches

1 stem 0 0 0 0 0 0

2 stems 1 1 1 1 1 1

3 stems 0 0 0 0 0 0

> 4 stems 2 2 2 2 2 2

All patches 3 3 3 3 3 3

Nonflowering patches

1 stem 5 (11.8) 6 (11.5) 4 (6.5) 4 (6.9) 2 (5.8) 10

[19.1,6.9] [15.3,7.6] [9.1,5.2] [10.4,5.3] [8.8,4.1]

2 stems 5 (9.7) 4 (6.4) 4 (5.7) 7 (10.2) 4 (8.8) 9

[17.6,6.8] [8.4,5.3] [6.9,5.0] [16.9,8.0] [15.8,5.9]

3 stems 2 (3.3) 3 (4.2) 3 (3.9) 3 (3.9) 2 (3.5) 3

[3.5,3.4] [4.6,3.9] [4.2,3.7] [4.2,3.7] [3.6,3.4]

> 4 stems 8 (11.6) 9 (11.5) 9 (10.9) 10 (11.8) 10 (14.7) 10

[20.8,9.0] [18.1,9.7] [16.9,9.5] [16.7,10.5] [20.9,12.0]

All patches 20 (30.4) 22 (30.8) 20 (30.4) 24 (31.1) 18 (30.0) 32

[32.0,29.0] [32.0,29.7] [32.0,29.0] [32.0,30.3] [32.0,28.3]

Time (min.) 103 104 91 140 128
